# PolysacDB: A Database of Microbial Polysaccharide Antigens and Their Antibodies

**DOI:** 10.1371/journal.pone.0034613

**Published:** 2012-04-11

**Authors:** Abhijit Aithal, Arun Sharma, Shilpy Joshi, Gajendra P. S. Raghava, Grish C. Varshney

**Affiliations:** 1 Cell Biology and Immunology Division, Institute of Microbial Technology, Chandigarh, India; 2 Bioinformatics Centre, Institute of Microbial Technology, Chandigarh, India; The Scripps Research Institute, United States of America

## Abstract

Vaccines based on microbial cell surface polysaccharides have long been considered as attractive means to control infectious diseases. To realize this goal, detailed systematic information about the antigenic polysaccharide is necessary. However, only a few databases that provide limited knowledge in this area are available. This paper describes PolysacDB, a manually curated database of antigenic polysaccharides. We collected and compiled comprehensive information from literature and web resources about antigenic polysaccharides of microbial origin. The current version of the database has 1,554 entries of 149 different antigenic polysaccharides from 347 different microbes. Each entry provides comprehensive information about an antigenic polysaccharide, i.e., its origin, function, protocols for its conjugation to carriers, antibodies produced, details of assay systems, specificities of antibodies, proposed epitopes involved and antibody utilities. For convenience to the user, we have integrated web interface for searching, advanced searching and browsing data in database. This database will be useful for researchers working on polysaccharide-based vaccines. It is freely available from the URL: http://crdd.osdd.net/raghava/polysacdb/.

## Introduction

Polysaccharides are composed of repeating units of a single sugar or an oligosaccharide moiety and may also contain non-carbohydrate constituents such as lipids or proteins [1]–[3]. They are often present on the cell surface of pathogenic microbes and have vital role in host-pathogen interactions. They are also excellent immunogens as they produce broad-spectrum immune responses which may lead to the protection of the host from the subsequent infection [4]. Hence microbial polysaccharides can be used to design vaccines to prevent the infectious diseases. In fact, a polysaccharide vaccine against *Streptococcus pneumoniae* has been licensed for use in humans [5]. Furthermore, with the increase in the incidence of antibiotic resistance which may limit our therapeutic choices in future, polysaccharide based vaccines could provide an attractive alternative as they are not modified by mutations. In this context, a database compiling all the antigenic polysaccharides becomes an urgent need as it will not only facilitate in designing newer polysaccharides-based vaccines against microbial pathogens but also augment our understanding about the host-pathogen interactions.

In the past, number of databases have been developed for designing peptide/protein-based vaccines such as IEDB [6], MHCBN [7], BCIPEP [8], AntiJen [9], PRRDB [10], SYFPEITHI [11] etc. Some databases such as IMGT [12] and DIGIT [13] give comprehensive information on immunoglobulin sequences. Previously, we had developed a database HaptenDB which provided comprehensive information on haptens [14]. Surprisingly, very few computational resources have been created in the area of carbohydrate/polysaccharide antigens. Immune Epitope database [IEDB] provides information on non-peptide epitopes (including carbohydrates) that are found in species ranging from humans to prokaryotic bacteria. However, this database does not provide any information on the characteristics of either antigens or antibodies. In order to provide a service to the scientific community involved in the development of carbohydrate or polysaccharides-based vaccines, we have made a systematic attempt to collect information about antigenic polysaccharides of microbial origin from literature and web-based resources. This information has been compiled in a database PolysacDB.

## Results and Discussion

This database was constructed mainly to create a resource that would facilitate easy retrieval of information which is otherwise scattered in literature. It is for the first time an attempt has been made to create a comprehensive database on antigenic polysaccharides of microbial origin. This database tries to bring the experimental data out into the open in a succinct and consolidated form. The data curation is completely manual which required considerable effort and time, as this involved delving deep into papers and deducing conclusions out of experimental data. We believe that such manual curation provides greater reliability and accuracy.

### Data collection and integration

The data collection was a baseline task for PolysacDB database and started with defining the full database schema. The data was collected from three primary sources viz. PubMed, BCSDB (Bacterial Carbohydrate Structure Database) and IEDB. Initially, the relevant papers were downloaded using appropriate keywords such as “microbe”, “polysaccharide”, “antibody” etc. They were then carefully scrutinized for relevant information. Only those papers revealing immunological data about the carbohydrate sub-structures on microbial cell surface were selected. Emphasis was especially given, to those where specific antibodies were used or generated. Papers that were limited in giving information only on polysaccharide structures or those giving information on polysaccharides from non-microbial species were removed. Thus among 600 peer-reviewed publications that were accessed, 400 were used for entering the data into the database. In addition to the published information, structures were derived from BCSDB database for a number of polysaccharides. Since each entry was based on the information derived from physical experiments, we have divided the information into two basic levels – one level describing the “experimental parameters” used and the other level describing the “results obtained”. Each entry in the database includes; i) general information like name, nature and functions of the polysaccharides; ii) protocols of immunization including carrier proteins, coupling protocols and assays and iii) name and nature of the antibodies, their specificities and cross-reactivities, proposed epitopes involved and antibody utilities. It was strived to give maximum information on the antibody cross-reactivity profiles and the epitopes involved. In order to provide comprehensive information, data in the database is linked to various databases such as BCSDB and IEDB.

### Database Schema, Statistics and Updating

**Figure 1:**
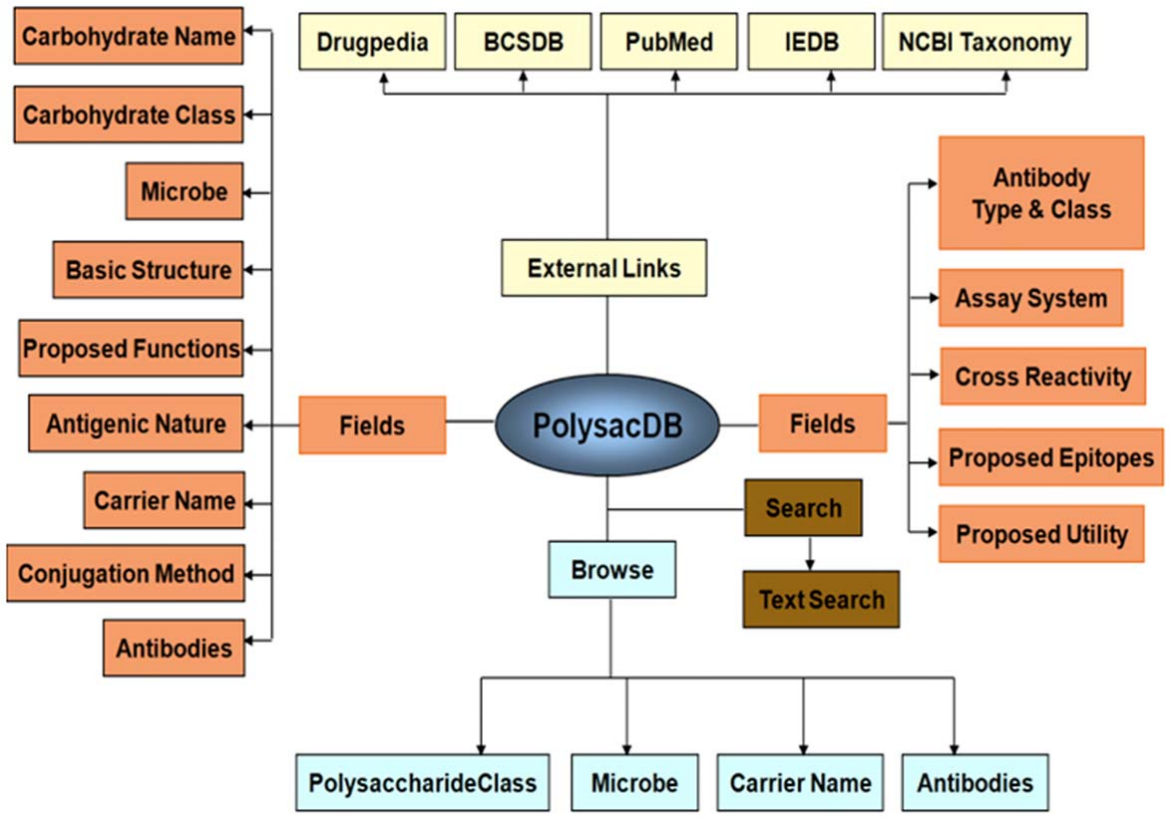
The complete database architecture showing its various components.

**Figure 2:**
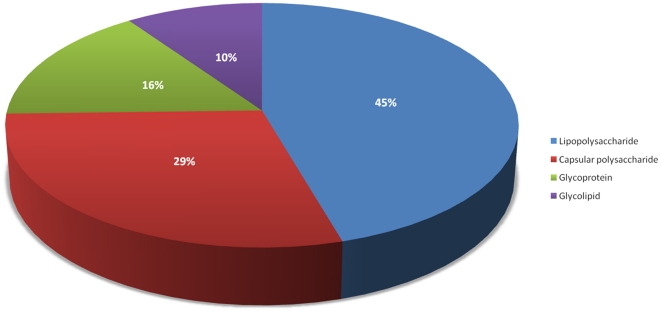
Relative prevalence of the various types of polysaccharides within PolysacDB.

Database statistics: The PolysacDB schema contains 18 unique fields, overall schema of the database is provided in Figure 1. The predominant antigenic polysaccharides that produce antibodies have been mainly classified into “Capsular Polysaccharide”, “Lipopolysaccharide”, “Glycolipids” and “Glycoproteins” classes. Such a classification would help in the general accessibility to the data and would create a new interface through which a user can search the database. Figure 2 shows the relative prevalence of these classes in PolysacDB. It could be seen from Figure 2 that most of the antigenic polysaccharides fall within the long chain polysaccharide classes such as “lipopolysaccharides” and “capsular polysaccharides” (74%), while a small portion falls within the “glycolipid” and “glycoprotein” classes (26%). This underscores the importance of chain length of a polysaccharide and the effect it has on its immunogenicity. Since the data in PolysacDB is purely based on experimental results, it is reasonable to assume that additional entries in future would continue to improve the scope of the database.

### Data updating tools

In order to maintain the usefulness of the database in the long run, it is necessary to create an efficient data updating system so that users all over the world will be able to add new entries as well as update the existing entries. In lieu of this, we have prepared a system that facilitates data updating via two different ways: first through the “Online Submission” webpage and second through DrugPedia. The “Online Submission” webpage provides the interface to add the data in the already prescribed field format in an intermediate table. The data from intermediate table will be validated by our team at an interval of two to three months and stored in main data storage table through a PHP script. DrugPedia is mainly conceived to facilitate modification of already existing data within the database. Each record obtained through search or browse provides links to DrugPedia where users may modify already existing data by either adding or deleting information. After proper validation by our expert team, the information from DrugPedia will be stored to main data storage table of PolysacDB database.

### User Interface

PolysacDB provides simple and user friendly interface to “search” and “browse” data. The “Search Option” allows user to search data on any field like keywords, microbe, polysaccharide name etc. Advanced Search option combines two types of queries, the name of the polysaccharide and the name of the microbe. For example, advance search on “lipopolysaccharide” (polysaccharides) and “*Vibrio cholerae*” (microbe) extract 59 entries, each describes the immunological properties of lipopolysaccharides present on the surface of *V. cholerae*. Search option provides a large number options: i) “Containing” and “Exact” type of search, ii) option for selecting specific fields to be searched and, iii) option for selecting specific fields to be displayed. Browse option provides a powerful interface to retrieve data without entering keywords. A total of four fields may be browsed directly viz. Polysaccharide classes, Microbe, Carrier Name and Antibodies. Each field further leads to the respective list of names that can be browsed to get the specific entries.

### Database utilities

An important utility of PolysacDB is that it can be used as a resource to investigate antigen-antibody interactions involving microbial polysaccharides by making use of specificities and avidities of the respective antibodies. For example, the cross-reactivity profile of an antibody, generated against a carbohydrate structure may indicate the presence of a similar structure, a common epitope, in two different species. For example, in the entries observed via the browse option within the *E. coli O18A* field, one can find that on immunizing mice with boiled *E. coli O18A* bacteria, antibodies with three types of specificities were obtained. One type reacted with only the homologous *E. coli* sps., the second type was cross-reactive with other *E. coli* sps. While the third type, although specific to the homologous species, also cross-reacted with *S. marcescens* 08 LPS. The epitope of the latter type of antibodies was found out to be N-acetyl-D-glucosamine. This may implicate that a structure similar to N-acetyl-D-glucosamine exists on the surface of *S. marcescens* [15]. Hence the database may be utilized to predict the cross-reactivity of anti-polysaccharide antibodies with particular microbes, based on the presence of similar polysaccharide epitopes. In other words, the database would be useful in predicting novel epitopes on polysacchaide backbones of different microbial species

PolysacDB gives information on the structures and functions of polysaccharides that are usually involved in basic house-keeping and virulence-associated functions within the microbe. It gives information on the methodologies used to conjugate polysaccharides to appropriate carriers for immunization purposes. Such protocols often dictate the nature of antibodies produced against a particular immunogen. It is also worthwhile to note that the majority of anti-polysaccharide antibodies have been produced using whole bacterial cells as immunizing material. A field describing the assay systems, such as ELISA, western blotting, immunofluorescence etc used for characterizing the antibodies is given. The epitopes involved have been enumerated and links to IEDB have been given wherever applicable. Since the surface polysaccharides play a vital role in the virulence of pathogens, the antibodies against these are often found to be protective and some of them are used as reliable therapeutic and detection reagents. The database provides information on these aspects in the antibody utility fields. PolysacDB also provides a platform that would facilitate users to order or request for procurement of antibodies from different organizations/sources.

### Limitations and future prospects

We have observed that information on antigenic polysaccharides is quite abundant, but scattered. Further literature search is required to expand the database and make it more comprehensive. Also, some of the data may seem redundant since many polysaccharides have more than one antibodies raised against it, but when we see the cross-reactivity and epitope fields of individual entries, it becomes clear that the entries are entirely different. From technical point of view, it is very difficult to maintain the quality of data since lots of special symbols are there in carbohydrate names and structures that creates problems while storing data in MySQL, though enough care was taken during data storage but still some efforts are required for proper data storage and display. In near future, we are hopeful of expanding the database both qualitatively as well as quantitatively to cover more information. The database will be further updated manually on a regular basis.

### Conclusions

PolysacDB provides online web tools that allow the users to retrieve and analyze the data. It includes tools: (1) for searching database using keywords with many options and (2) for browsing the database on polysaccharide names and microbes. It also shows specific details on aspects such as structure and functions of the polysaccharides, conjugation protocols to carriers and characteristics and utilities of the antibodies. In addition, it provides internal hyperlinks to display detailed information and external hyperlinks to other databases. In future, PolysacDB will be further improved by providing detailed two-dimensional structures of polysaccharides along with the spatial conformation and location of the epitopes within such structures. We believe that PolysacDB will facilitate the open source knowledge distribution of polysaccharide antigens and contribute to more detailed understanding of their immunogenicity and antigenicity.

## Materials and Methods

### Web Interface and Application

The whole database is running under LAMP (Linux-Apache-Mysql-PHP) Server technology, integration of four open source softwares. PHP, HTML and CSS technologies have been used to build the dynamic web interface. MySQL, a relational database management system (RDBMS), works at the backend and provides commands to retrieve stored antigenic carbohydrate data. PHP, a server side scripting language, provides interface and functions to fetch data from database. The whole software system runs on IBM SAS x3800 machine under Red Hat Enterprise Linux 5 environment using Apache httpd server. PHP and MySQL combination is quite efficient and powerful for database management.
